# Cross-border malaria in Northern Brazil

**DOI:** 10.1186/s12936-021-03668-4

**Published:** 2021-03-06

**Authors:** Nicholas J. Arisco, Cassio Peterka, Marcia C. Castro

**Affiliations:** 1 Department of Global Health and Population, Harvard T.H. Chan School of Public Health, 655 Huntington Avenue, Building 1, Room 1002A, Boston, MA 02115 USA; 2 Diretoria de Vigilancia Epidemiológica, Secretaria de Estado de Saúde Do DF, Brasília, DF 70390-125 Brazil

**Keywords:** Cross-border malaria, Malaria elimination, Imported malaria

## Abstract

**Background:**

Cross-border malaria is a major barrier to elimination efforts. Along the Venezuela-Brazil-Guyana border, intense human mobility fueled primarily by a humanitarian crisis and illegal gold mining activities has increased the occurrence of cross-border cases in Brazil. Roraima, a Brazilian state situated between Venezuela and Guyana, bears the greatest burden. This study analyses the current cross-border malaria epidemiology in Northern Brazil between the years 2007 and 2018.

**Methods:**

De-identified data on reported malaria cases in Brazil were obtained from the Malaria Epidemiological Surveillance Information System for the years 2007 to 2018. Pearson’s Chi-Square test of differences was utilized to assess differences between characteristics of cross-border cases originating from Venezuela and Guyana, and between border and transnational cases. A logistic regression model was used to predict imported status of cases.

**Results:**

Cross-border cases from Venezuela and Guyana made up the majority of border and transnational cases since 2012, and Roraima remained the largest receiving state for cross-border cases over this period. There were significant differences in the profiles of border and transnational cases originating from Venezuela and Guyana, including type of movement and nationality of patients. Logistic regression results demonstrated Venezuelan and Guyanese nationals, Brazilian miners, males, and individuals of working age had heightened odds of being an imported case. Furthermore, Venezuelan citizens had heightened odds of seeking care in municipalities adjacent Venezuela, rather than transnational municipalities.

**Conclusions:**

Cross-border malaria contributes to the malaria burden at the Venezuela-Guyana-Brazil border. The identification of distinct profiles of case importation provides evidence on the need to strengthen surveillance at border areas, and to deploy tailored strategies that recognize different mobility routes, such as the movement of refuge-seeking individuals and of Brazilians working in mining.

## Background

Cross-border malaria is a major barrier to elimination efforts [1]. It can be of two types. First, transnational malaria, defined as an internationally imported case to a location not within a border area (sending and receiving countries may or may not be adjacent). Second, border malaria, defined as an internationally imported case across or along borders between countries sharing a land border (the border region can extend up through the adjacent administrative areas along the international border, or up to a specified distance from an international border) [2]. As cross-border malaria cannot be solved unilaterally, many cross-country collaborations have been established to enhance surveillance and control at borders [3, 4]. Yet, those collaborations may be difficult or impracticable when countries differ widely in their progress and commitment toward malaria elimination when one of the countries faces civil or political unrest, and when national protocols for diagnosis, treatment, and control are distinct [1].

Cross-border malaria presents many challenges, such as the remoteness of border regions, often with limited access to health services; the varied nature of population mobility (seasonal, illegal, driven by economic opportunities, or resulting from a humanitarian crisis); the difficulty in devising surveillance systems for mobile populations; and the incomplete adherence to medication that can trigger drug resistance [2]. Countries close to malaria elimination may have the last remaining cases occurring along international borders (e.g., Bhutan and India), and cross-border malaria is often a threat to countries that have eliminated malaria (e.g., the resurgence in Costa Rica and Swaziland) [1] or are close to elimination (e.g., Suriname [5, 6]).

In the Americas, malaria epidemiology has seen major changes in the past two decades. From 2000 to 2015, malaria cases declined 61.2% in Latin America, Brazil launched a *Plasmodium falciparum* elimination plan in 2015 [7], Paraguay and Argentina were certified malaria-free in 2018 and 2019, respectively [8], and El Salvador has reported zero indigenous cases since 2017 and in 2020 applied for malaria-free certification [9, 10]. However, during the same period, malaria cases increased by 359% in Venezuela and reached 411,586 in 2017 (53% of the cases in the Americas) [11–13]. After recording the lowest level in 36 years in 2016, malaria cases increased by 56.9% in Brazil in 2017 and again by 2.21% in 2018 [7]. In Guyana, malaria cases increased 25.5% in the same period, though the total number of confirmed cases annually remains under 15,000 [14].

This evolving epidemiology in the Americas brought attention to cross-border malaria along the Venezuela-Brazil-Guyana border. Following political changes that started in 2013, and an economic crisis that escalated starting in 2016, Venezuela faced major challenges, including in healthcare, and protests erupted. It is estimated that 4.6 million people had fled Venezuela by later 2020 [15]. The crisis also fueled an onrush of migrants to gold mines in Bolivar State (bordering Brazil, and where 70–80% of malaria cases are concentrated), boosting incidence locally [13], followed by a spillover to other areas in Venezuela and across international borders. Guyana, where artemisinin resistance is suspected to be emerging [16], has been a hotspot for illegal gold-mining, and economic activity that has been historically associated with human migration and malaria transmission in the Amazon. The type of gold extraction in the region contributes to the creation of puddles of stagnant water that favour mosquito breeding [17–21]. Reduced access to diagnosis, poor quality treatment, and minimal protection against mosquito biting has increased the vulnerability of miners to malaria. Therefore, the Venezuela-Brazil-Guyana border witnesses intense human mobility fueled primarily by a humanitarian crisis and illegal gold mining activities.

Roraima, a Brazilian state situated between Venezuela and Guyana, bears the greatest burden among Brazilian states [22]. Since the beginning of 2016, cross-border cases imported into Roraima have increased to nearly 500 infections per month, from less than 100 in 2015. Two municipalities in Roraima, namely Boa Vista (the capital) and Pacaraima (bordering Venezuela), have recorded more than half of all cross-border malaria cases into Brazil between 2007 and 2018. Major concerns emerged among cases imported from French Guiana [20], Suriname [6], Guyana [7, 12], and Venezuela [13, 23, 24].

This study is a comprehensive overview of the current cross-border malaria epidemiology in Northern Brazil, specifically along the Venezuela and Guyana borders, considering the years 2007 to 2018. Spatial and temporal patterns in both border and transnational malaria are characterized, contrasting demographic and epidemiological profiles of malaria importation from Guyana and Venezuela.

## Methods

### Study location

**Fig. 1:**
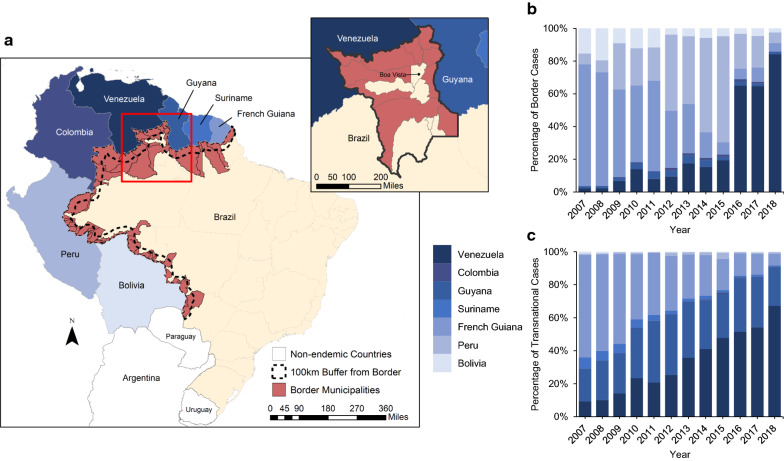
Border area in Brazil, study region, and cross-border malaria cases (2007–2018). **a** Brazil, border countries, border area according to the WHO definition, and 100 km buffer area from the border of malaria-endemic countries; insert map shows the state of Roraima. **b** Percentage of border malaria cases reported in the border municipalities by country origin and year. **c** Percentage of transnational malaria cases reported in the Amazon region by country of origin (considering only the bordering countries) and year

Brazil is divided into 5570 municipalities, and 756 of those are in the Brazilian Amazon where 99.5% of national malaria cases are reported. Along the Amazon region, 53 Brazilian municipalities share physical borders with six malaria-endemic countries; those are denoted border municipalities, and comprise the border region (Fig. 1). Malaria cases imported from adjacent countries to those 53 municipalities were defined as border malaria cases. Imported cases to any municipality other than the border ones, no matter the country of origin, were defined as transnational cases. Combined, they represent the number of cross-border malaria cases reported in Brazil.

The analysis focuses on the Venezuela-Brazil-Guyana border, particularly in the state of Roraima, which is divided into 15 municipalities, five of which share a border with Guyana, and five of which share a border with Venezuela (Fig. 1). The capital of Roraima, Boa Vista, had a population of 375,374 people in 2018 (65.1% of the state population). Between 2007 and 2018, 33% of all cross-border cases in Brazil were notified in Roraima.

### Data

De-identified data on reported malaria cases in Brazil were obtained from the Malaria Epidemiological Surveillance Information System (SIVEP) for the years 2007 to 2018. In Brazil, all cases are confirmed by microscopy or a rapid test (no case is reported based solely on clinical manifestations). The following variables were extracted: date of notification (MM/DD/YY); type of diagnosis (microscopy or rapid test); parasite type; locality (smaller areas defined by the National Malaria Control Program—NMCP) and municipality where the case was reported; locality, municipality, and country where the infection was likely to have occurred (if imported), given the travel history information; municipality of residence; age; gender; occupation; and type of case detection (active, passive). Based on those variables, imported malaria refers to a case whose most likely place of infection is different than the place where it was diagnosed [25–27]. Here, only consider cross-border malaria cases are considered and, therefore, importation between Brazilian localities was not analysed.

SIVEP includes cases reported through both passive and active detection (about 75% of cases are passively detected). One could argue that some infections may be missed due to precarious access to a health facility. However, in Brazil, this is expected to be minimal among individuals that are symptomatic because: (i) health care is universal and freely available, and malaria drugs are only available through the government (they are not sold in drugstores), so people traditionally search for care in the vast network of health posts and laboratories; and (ii) active case detection is regularly conducted by community agents in isolated areas with difficult access (e.g., riverine communities).

Map files were obtained from the Brazilian Institute of Geography and Statistics, projected using SIRGAS 2000-Mercator, and mapped in ArcGIS 10.6 (ESRI; Redlands, CA).

### Analytical methods

Characteristics of cross-border cases were summarized based on SIVEP variables. Pearson’s Chi-Square test of differences was utilized to assess differences between characteristics of cross-border cases originating from Venezuela and Guyana, and between border and transnational cases. Temporal trends were described from 2007 to 2018, considering cases reported weekly. A 60-day moving average was used to smooth the time series of malaria cases.

Since 2016 marked the intensification of the crisis in Venezuela, individual records of malaria cases aggregated for the period from 2016 to 2018 were used to assess factors associated with the occurrence of cross-border malaria in Roraima (n = 58,536). A total of 4,018 observations were missing data on occupation and were thus excluded from the regression model, amounting to a final sample size of 54,518 individuals. A logistic regression model with an indicator for imported cases as the outcome was used, including the following explanatory variables: nationality (Brazilian, Venezuelan, Guyanese, other), cross-border malaria type (transnational, border), gender (male, female), age group (< 5 years, 5–15, 16–24, 25–40, 41–64, 65 +), activity (agriculture, domestic, forestry, hunter/fisherman, miner, tourist, travelling, and other), parasite species (*Plasmodium vivax*, *Plasmodium falciparum*, mixed/other), type of detection (passive, active), and an interaction term between nationality and cross-border malaria type. The interaction term was added to capture possible differences in malaria importation driven by a humanitarian crisis (Venezuela) and economic activity (Guyana). The goodness of fit was assessed the Akaike information criterion, which presented a lower value for the model including the interaction signifying better model fit. Models were run in R version 3.4.2 [28]; data and trends were assessed using the dplyr package, and graphics were made using the ggplot2 package.

## Results

Between 2007 and 2016, the number of malaria cases reported in Brazil dropped 72.9%; the decline for autochthonous cases was 73.4%, and for cross-border cases it was 47.6% (Table 1). However, between 2016 and 2018, autochthonous and cross-border cases increased by 60.9% and 147.0%, respectively. While cross-border cases represented, on average, around 3% of all reported cases in Brazil, the origin of those cases changed between 2007 and 2018, and four issues stand out (Table 1, Fig. 1). First, less than 1% of cross-border cases originate in countries that do not share a border with the Brazilian Amazon. Second, about 41% of the cross-border cases, on average, were border cases, and thus were reported in Brazilian municipalities that share an international border. Third, the majority of cross-border cases originated from French Guiana in the first half of the study period (2007–2012), with a peak of 68% in 2007, while Venezuela was the main source in the second half of the period (2013–2018), with a peak of 75% in 2018. Fourth, considering the types of cross-border malaria (i) French Guiana was the most important source of both border and transnational cases between 2007 to 2011, (ii) the majority of border cases between 2012 and 2015 originated from Peru, (iii) Guyana was the main source of transnational cases from 2012 to 2014, and (iv) Venezuela was the origin of the majority of border and transnational cases from 2016 to 2018 (in 2018, 67% and 84% of transnational and border malaria cases, respectively, originated from Venezuela). Roraima state was the recipient of most cross-border cases (70.7%), especially those originating from Venezuela (91.4%) and Guyana (67%). Among cross-border cases from Venezuela, 50.1% were notified in border municipalities, against only 4.8% of those from Guyana.

**Table 1:** Annual number of total, autochthonous, and cross-border malaria cases in Brazil. Cross-border cases reported in the Amazon region are detailed by type and by country of origin (considering only countries that share an international border with the Amazon)

Category	Year											
	2007	2008	2009	2010	2011	2012	2013	2014	2015	2016	2017	2018
Malaria cases in Brazil												
Total	558,598	376,977	370,558	414,849	317,207	280,995	205,432	163,697	165,828	151,622	237,885	243,143
Autochthonous	547,405	369,013	361,810	404,761	310,732	272,576	195,031	158,015	160,159	145,760	231,897	234,525
Cross-border	11,193	7964	8748	10,088	6475	8419	10,401	5682	669	5862	5988	8618
Total Cross-border cases in the Amazon (by country of origin)												
Venezuela	648	534	1007	1994	1070	1570	3042	1811	1845	3358	3491	6,61
Border	122	76	202	515	166	321	618	301	577	1670	1629	3370
Transnational	526	458	805	1,479	904	1249	2424	1510	1268	1688	1862	3091
Guyana	1189	1121	1416	2051	1713	1926	2457	1172	775	1130	1099	1139
Border	72	47	53	133	90	126	176	80	66	61	47	53
Transnational	1117	1074	1363	1918	1623	1800	2281	1092	709	1069	1052	1086
Colombia	5	7	29	36	17	67	60	43	61	50	33	18
Border	2	2	15	20	8	56	43	28	42	37	24	10
Transnational	3	5	14	16	9	11	17	15	19	13	9	8
Suriname	392	260	330	320	155	119	146	84	42	38	38	35
Border	1	2	2	–	2	1	5	–	1	3	–	1
Transnational	391	258	328	320	153	118	141	84	41	35	38	34
French Guiana	7550	4999	4695	4224	2792	2829	2867	1207	717	600	650	538
Border	4047	2364	1596	1727	1151	1189	1063	305	220	160	221	211
Transnational	3503	2635	3099	2497	1641	1640	1804	902	497	440	429	327
Peru	401	280	904	911	448	1693	1563	1198	2046	572	511	299
Border	354	252	847	842	422	1590	1468	1131	1942	549	484	259
Transnational	47	28	57	69	26	103	95	67	104	23	27	40
Bolivia	901	709	290	475	250	153	186	123	157	96	139	113
Border	838	665	271	445	243	130	170	114	143	85	120	101
Transnational	63	44	19	30	7	23	16	9	14	11	19	12

**Fig. 2:**
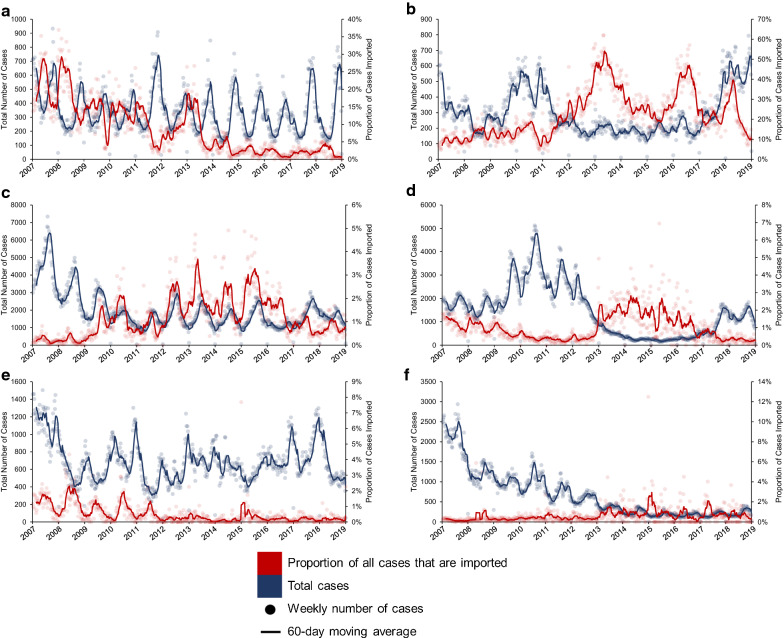
Total reported malaria cases per week in each state, and proportion of cases imported into states, 2007–18. Each panel represents a state along an international border in Brazil: **a** Amapá, **b** Roraima, **c** Amazonas, **d** Pará, **e** Acre, and **f** Rondônia. Scales in the primary and secondary Y-axis are not uniform

**Fig. 3:**
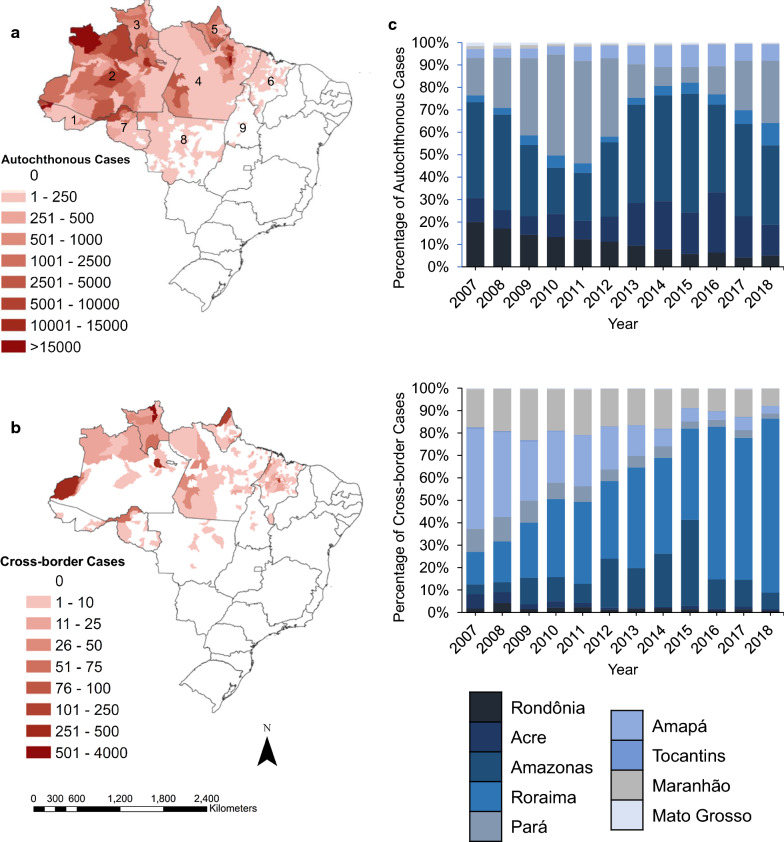
Autochthonous and border cases in Brazil by municipality (aggregate 2016–2018) and state (yearly). **a** Sum of all autochthonous malaria cases in each municipality in Brazil from 2016 to 2018. **b** Sum of all cross-border malaria cases in each municipality in Brazil from 2016 to 2018. **c** Percentage of autochthonous malaria cases (top) and cross-border malaria cases (bottom) that were reported in Brazil, separated by state and plotted across years. Each state is depicted by a unique colour

Among states in the Brazilian Amazon that share an international border, the percentage contribution of cross-border malaria cases among all cases reported varied spatially and temporally. Between 2007 and 2018, Roraima had the highest share of cross-border cases among all malaria cases reported in the state, while in Amapá the contribution never surpassed 1% (Fig. 2). However, during the same period, the distribution of autochthonous (Fig. 3a) and cross-border (Fig. 3b) cases largely varied by municipality.

**Fig. 4:**
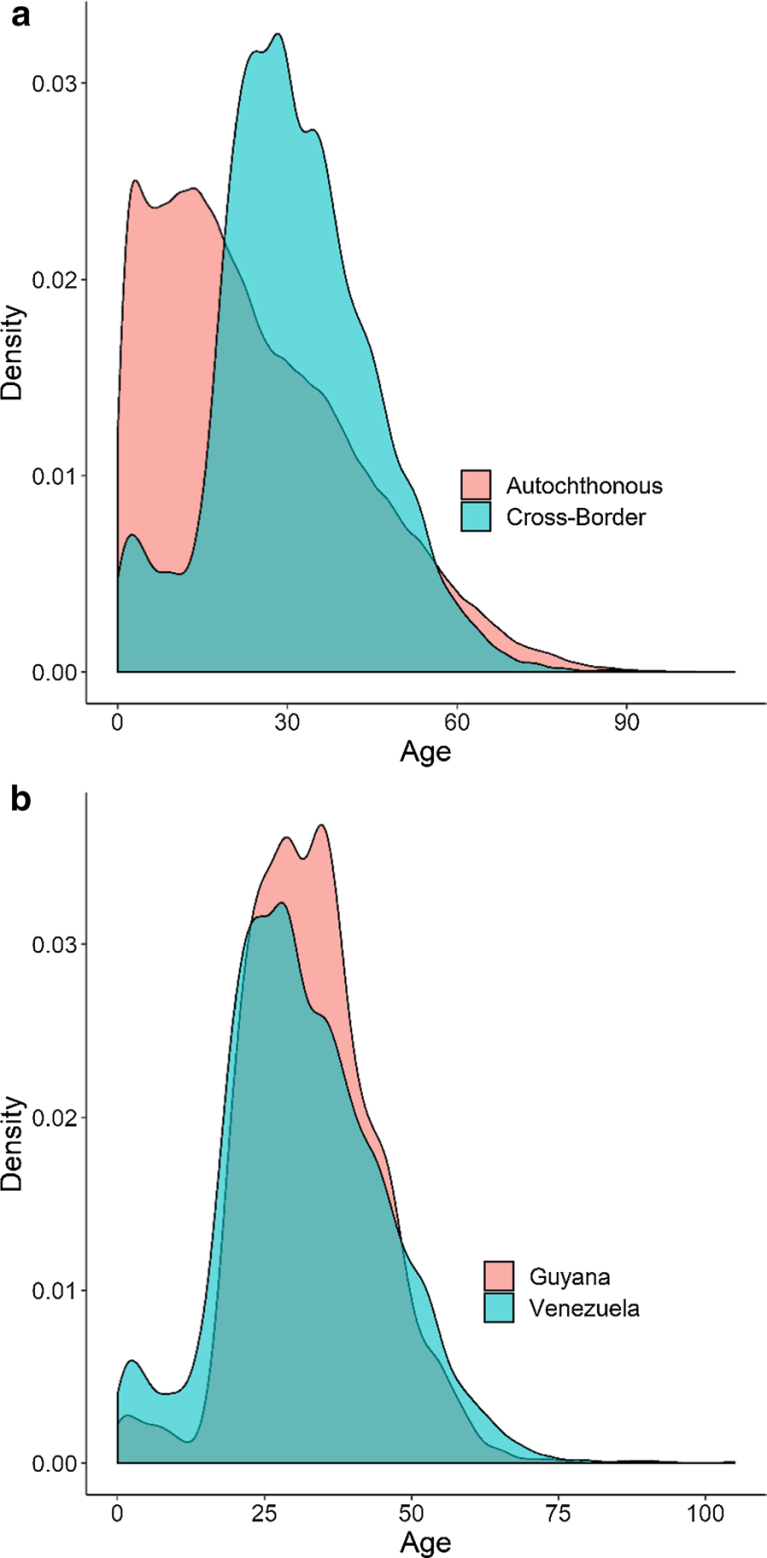
Proportion of malaria cases comprising each age. Density plots of age distribution of cases broken down by case type: **a** autochthonous and cross-border cases, **b** cross-border cases originated from Venezuela and Guyana

Considering the years 2016 and 2018, characteristics of autochthonous and cross-border border malaria cases showed statistically significant differences (Table 2). The number of Venezuelan citizens infected abroad was more than 200 times the number of those infected in Brazil. Mining was by far the dominant occupation among cross-border cases, while agriculture was the most common among autochthonous cases. The age distribution of autochthonous and cross-border cases showed distinct patterns (Fig. 4a). Autochthonous cases peaked early, had a median age of 21 years, and consistently declined across adult ages. Cross-border cases had a delayed pattern, with a median age of 30, and two peaks: a small one at young children’s ages, and a more pronounced one around age 30.

**Table 2:** Characteristics of autochthonous and cross-border malaria cases, Brazil, 2016–18

Variable	Total autoch-thonous cases	Total cross-border cases	Χ^2^ p-value	Cross-border cases Venezuela	Cross-border cases Guyana	Cross-border cases Other Countries	Χ^2^ p-value
Sex			< 0.001				< 0.001
Female	245,245	6619		4435	1034	1150	
Male	366,783	13,849		8875	2334	2640	
Age group			< 0.001				< 0.001
Under 5	67,788	739		424	54	261	
5 to 15	162,305	1239		696	58	485	
16 to 24	116,944	4442		3013	656	773	
25 to 40	146,951	9186		5827	1830	1529	
41 to 64	102,175	639		3192	754	693	
Over 65	15,865	223		158	15	50	
Nationality			< 0.001				< 0.001
Brazil	611,966	13,467		7157	3331	2979	
Venezuela	30	6157		6149	6	2	
Guyana	10	32		3	29	–	
Others	22	812		1	1	810	
Occupation			< 0.001				< 0.001
Agriculture	137,227	1166		316	131	719	
Domestic	59,161	738		459	67	212	
Forestry	5713	154		57	15	82	
Hunter/Fisherman	16,076	152		36	3	113	
Miner	16,392	13,485		9789	2518	1178	
Tourist	3137	161		112	26	23	
Travelling	8506	454		296	48	110	
Other	365,816	4158		2245	559	1354	
Detection method			< 0.001				< 0.001
Active	153,133	911		230	145	536	
Passive	457,235	19,520		13,051	3216	3253	
Parasite species			< 0.001				< 0.001
*P. falciparum*	40,348	3349		2513	416	420	
*P. vivax*	547,714	15,395		9576	2701	3118	
Mixed/other	23,966	1724		1,221	250	253	
State			< 0.001				< 0.001
Acre	110,081	182		2	–	180	
Amapá	46,965	839		9	2	828	
Amazonas	248,386	2111		496	107	1,508	
Pará	129,519	587		194	203	190	
Rondônia	30,527	207		6	11	190	
Roraima	42,201	14,468		12,166	2257	45	
Others	4349	2074		437	788	849	
Type of cross-border			< 0.001				< 0.001
Border	246,471	9098		77	2523	77	
Transnational	365,557	11,370		3290	1267	3290

Similarly, cross-border cases originating from Venezuela, Guyana, and other countries differed in the composition of every categorical variable that was analysed (Table 2). An important difference was in the nationality of cases: 98.9% of cross-border cases originating from Guyana were Brazilian nationals, while slightly over half (53.8%) originating from Venezuela were Brazilians. The type of occupation of most cross-border cases from Venezuela and Guyana was mining, while less than a third of cases originating in other countries declared this type of activity. With regards to parasite type, 75.2% of all cross-border cases were *P. vivax*, which is lower than the share of *P. vivax* among all malaria cases recorded in Brazil during the same period, 89.02%. Of the cross-border cases originating from Venezuela and Guyana, the percentage of *P. falciparum* cases were, respectively, 18.9% and 12.4%. Most cross-border malaria cases were passively detected (95.3%). With regards to age, 66.6% of cross-border cases were between 16 and 40 years; 73.8% of those originating from Guyana were in that age range. Also, 8.4% of the cross-border cases from Venezuela had ages under 15 years, against only 3.3% of those from Guyana (Fig. 4b).

Characteristics of malaria cases also varied by type of cross-border case, considering Venezuela and Guyana aggregated from 2016 to 2018 (Table 3). In the case of Venezuela, three important issues stand out. First, most transnational cases (95.6%) were Brazilians, while 87.8% of the border cases were Venezuelans. Second, 81.2% of border cases were individuals working on mining activities, against 65.8% of the transnational cases. Third, while the share of *P. vivax* cases was similar, 21.2% and 16.7% of the border and transnational cases, respectively, were *P. falciparum*. Among cross-border cases from Guyana, two distinctions are important. First, and in contrast with Venezuela, working on mining was more prevalent among transnational cases from Guyana (78.8%, against 55.3% in border cases). Second, 18.1% of border cases originating from Guyana were detected via active surveillance, a higher percentage than transnational cases (3.9%). Comparatively, 1% of the border cases and 2.6% of transnational cases with infections originating from Venezuela were diagnosed through active case detection.

**Table 3:** Characteristics of cross-border malaria cases originating from Venezuela and Guyana into Brazil, by type, 2016–18

Variable	From Venezuela			From Guyana		
	Border	Transna-tional	χ^2^ p-value	Border	Transna-tional	χ^2^ p-value
Sex			0.012			0.126
Female	2234	2201		17	1016	
Male	4264	4611		60	2274	
Age group			< 0.001			0.071
Under 5	226	198		1	53	
5 to 15	442	254		2	56	
16 to 24	1524	1,489		21	635	
25 to 40	2666	3,161		29	1801	
41 to 64	1533	1659		23	731	
Over 65	107	51		1	14	
Nationality			< 0.001			< 0.001
Brazil	671	6486		73	3,258	
Venezuela	5827	322		0	6	
G uyana	0	3		4	25	
Others	0	1		0	1	
Occupation			< 0.001			< 0.001
Agriculture	126	190		9	122	
Domestic	370	89		1	66	
Forestry	13	44		1	14	
Hunter/fisherman	20	16		0	3	
Miner	5370	4419		46	2472	
Tourist	21	91		1	25	
Travelling	87	209		2	46	
Other	491	1754		36	542	
Detection method			< 0.001			< 0.001
Active detection	57	171		18	127	
Passive detection	6439	6612		81	3136	
Parasite species			< 0.001			0.930
*P. falciparum*	1384	1129		9	407	
*P. vivax*	4606	4970		63	2638	
Mixed/other	508	713		5	245	
State			< 0.001			< 0.001
Acre	0	2		0	0	
Amapá	0	7		0	2	
Amazonas	28	468		0	107	
Pará	0	186		0	203	
Rondônia	0	6		0	11	
Roraima	6470	5696		77	2179	
Others	0	437		0	788	

There were 58,532 cases of malaria notified in the state of Roraima between 2016 and 2018. Of these, 14,467 were cross-border cases (99.7% from Venezuela and Guyana). Table 4 shows the results of the multivariable model. All variables, except The *P. falciparum* category, and the interactions between Guyanese nationality and the border indicator were statistically significant with p < 0.05. Guyanese and Venezuelan nationals had a 49.03-fold (95% CI: 7.95, 302.20) and 233.76-fold (95% CI: 109.41, 499.43) odds of being an imported case, respectively, as compared to individuals of Brazilian nationality. The estimate for cases with nationalities other than these three as compared to Brazil was high, but unstable and not statistically significant. Cases notifying in border municipalities had 0.56-fold (95% CI: 0.50, 0.62) odds of being imported as compared to transnational municipalities. However, the fitted interaction term demonstrates that among cases notifying at the border, imported cases have a 9.48-fold higher odds of being Venezuelan nationals as compared to cases notifying transnationally, above their baseline estimate of 233.76.

**Table 4:** Correlates of cross-border malaria reported in Roraima and originating from Venezuela and Guyana, 2016–2018

Variable name	Odds ratio	Lower 95% CI	Upper 95% CI	P-value
Intercept	0.07	0.05	0.09	< 0.001
Nationality (Ref. = Brazilian)				
Guyanese	49.03	7.95	302.20	< 0.001
Venezuelan	233.76	109.41	499.43	
Type of cross-border (Ref. = Transnational)				
Border	0.56	0.50	0.62	< 0.001
Sex (Ref. Female)				
Male	0.90	0.82	0.97	0.010
Age group (Ref. ≤ 5 years)				
5 to 15	0.57	0.46	0.71	< 0.001
16 to 24	2.28	1.89	2.75	< 0.001
25 to 40	2.63	2.20	3.16	< 0.001
41 to 64	1.78	1.47	2.15	< 0.001
65+	0.67	0.46	0.98	0.038
Occupation (Ref. = Other)				
Agriculture	0.22	0.19	0.25	< 0.001
Domestic	0.58	0.48	0.71	< 0.001
Forestry	2.66	1.95	3.62	< 0.001
Hunting/Fishing	0.10	0.06	0.16	< 0.001
Mining	55.76	50.59	61.46	< 0.001
Tourism	4.60	3.50	6.04	< 0.001
Traveling	1.62	1.37	1.92	< 0.001
Parasite (Ref. = Mixed/Other)				
*P. falciparum*	0.93	0.73	1.19	< 0.560
*P. vivax*	0.18	0.15	0.22	< 0.001
Interaction terms				
Guyana:Border	0.74	0.09	6.25	0.783
Venezuela:Border	9.48	3.68	24.43	< 0.001
Detection type (Ref. = active)				
Passive	5.30	4.24	6.61	< 0.001

Other variables associated with an increased odds of being an imported case were being between the ages of 16 and 64, being infected with *P. falciparum*, and having either forestry, mining, tourism, or travelling as an occupation. Variables associated with decreased odds of being an imported case were being between the ages of 5 and 15 or 65 + , being infected with *P. vivax*, and working in agriculture, living domestically, or being either a hunter or fisherman.

## Discussion

This study comprehensively analysed the spatial and temporal dynamics of cross-border malaria in northern Brazil, particularly along the Brazil-Venezuela-Guyana border, and identified the correlates of cross-border cases in Roraima. Results show that cross-border cases from Venezuela and Guyana made up the majority of border and transnational cases since 2012, and that Roraima has remained the largest receiving state for cross-border cases over this period. There were significant differences in the profiles of cross-border and autochthonous cases as well as border and transnational cases originating from Venezuela and Guyana.

The results of this study bring about four important points relevant to malaria control and elimination in Brazil. First, border areas are highly vulnerable to remaining malaria hotspots despite country-wide elimination efforts. Since 2016, cross-border cases in the state of Roraima have been on the rise, mainly originating in Venezuela and Guyana. Within the state of Roraima, border municipalities tend to be isolated, with weaker surveillance and treatment capabilities than more internal municipalities [2, 22]. While active case detection is higher in border municipalities, the share of cases actively detected is still quite low. Also, civil strife and humanitarian crisis, resulting in moment across international borders, may happen suddenly. In the state of Roraima, cases imported from Venezuela increased quickly in a short period as the crisis in the country intensified [13, 22, 29]. The combination of limited resources and governance, unpredictable mobility, and some economic activities (such as mining) situate border regions as areas vulnerable to outbreaks of malaria that may challenge country-wide elimination efforts. This is true for other diseases; in 2018 Roraima reported over 300 measles cases (a disease that had been eliminated), traced back to predominantly Venezuelan migrants [22, 30]. The example of measles demonstrates the fragility of sustained disease elimination in Brazil’s border regions and illuminates the importance of understanding dynamics at the border and stratifying interventions accordingly [31].

Second, these results demonstrate the importance of recognizing vulnerable border areas and implementing surveillance as an intervention in line with the World Health Organization (WHO) Global Technical Strategy for malaria [32]. The third pillar of the WHO Global Technical Strategy for malaria 2016–2030 focuses on strengthening surveillance efforts to become a core intervention, as data collection and detection of cases is of paramount importance when planning the stratification of control efforts and resource distribution. Data-driven approaches to malaria stratification, particularly those that include cross-country collaboration with intense surveillance, are effective in combatting cross-border malaria [33–35], although cross-country collaboration may be impracticable in some cases due to incongruent surveillance efforts or poor international relations across country borders [4]. Genetic and molecular techniques have the potential to monitor drug-resistant malaria parasites entering from neighboring countries [36]. This is particularly important as artemisinin resistance is suspected in Guyana [37, 38]. Implementation of genetic and molecular screening techniques in regions of the world with the infrastructure and economic capabilities of scaling these methods has proven invaluable when transmission is low and elimination is in sight [39–41]. These methods may prove useful in certain contexts within the Brazilian Amazon, particularly in the state of Roraima, for the monitoring of drug resistance.

Third, the importance of understanding the profiles of different types of cross-border malaria cases was demonstrated. As opposed to the Venezuelan case, most of the cross-border malaria cases originating in Guyana are Brazilians who cross the border back-and-forth to work on gold mining. This dynamic is supported in the literature, and the results from this study corroborate the importance of Boa Vista as a hub for mobile mining populations seeking medical care [42]. Ultimately, the response to cross-border cases is inextricably linked to the demographic profiles of each population. For example, Venezuelan migrants seeking medical care and refuge in Brazil may be more likely to remain in the Brazilian Amazon if they receive refugee status [13, 22, 29]. Strengthening surveillance at the border among Venezuelan migrants, as well as treatment capacity along common routes of travel for these groups, contrast the need for targeted care, prophylactic measures, and active surveillance of Brazilian miners working in Guyana and seeking care in Boa Vista.

**Fig. 5:**
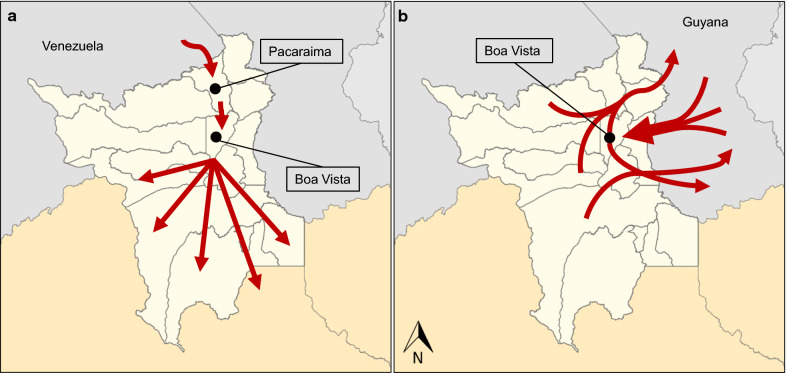
Likely routes of malaria cases imported from Venezuela and Guyana. **a** Represents the pattern of migration from Venezuelan migrants, while **b** represents the pattern of migration from Guyanese migrants. Red arrows signify hypothesized migration flow patterns

Two distinct movement patterns of cross-border cases travelling from Venezuela and Guyana into Roraima were hypothesized (Fig. 5). In the case of Venezuela (Fig. 5a), individuals fleeing the country cross the border into Roraima, making their first point of contact in the municipality of Pacaraima [22]. While many may settle there, others seek better conditions or apply for refugee status, eventually getting settled in other municipalities in Brazil [43]. In the case of Guyana (Fig. 5b), cross-border cases are predominantly Brazilian nationals travelling for economic opportunity (mainly gold mining, mostly illegal) [44]. These movements are likely to occur repeatedly and present a challenge to both Brazil and Guyana [42, 44]. In Guyana, 94% of malaria cases reported occurred in major gold mining regions, and the malaria scenario in Venezuela demonstrates the potential for small, isolated, malaria-dense populations with minimal resources to spur outbreaks of the disease and reverse progress toward elimination.

Fourth, the isolation of border regions and caseloads depending on two country’s malaria policies rather than one present major challenges for the implementation of control measures. The border of Roraima with Venezuela and Guyana are forested and porous. The potential for these regions to sustain malaria control without the ability to directly target at-risk populations may come from the introduction of novel interventions. Currently, Brazilians mining in Guyana travel back to Boa Vista to seek health care. Prophylactic interventions and more readily accessible treatment can be distributed far closer to gold mining sites, allowing individuals to increase economic productivity while preventing further infections and risk of cross-border cases. Malakit, a personal prophylactic and treatment kit, is one such example of an intervention that brings necessary services closer to the at-risk populations [45]. The borders of Venezuela and Guyana may be locations where interventions like Malakit, coupled with widespread active surveillance, could be highly effective. However, maximizing the efficiency and utility of this intervention will depend on knowledge of specific travel routes taken between neighbouring countries by high-risk individuals. Furthermore, efficacy will depend on neighbouring countries’ ability to collaborate. If efforts to curb malaria transmission are uneven across international borders, malaria elimination efforts are threatened [2].

This study has some limitations. Asymptomatic malaria infections are largely not captured by the NMCP in Brazil (similar to other countries), as individuals without symptoms do not seek medical care. Also, foreigners may report their nationality incorrectly (as Brazilians) when they seek medical care. Both limitations imply that these study results could be an underestimation of cross-border cases. Yet, data utilized in the analysis are the most comprehensive available.

## Conclusion

Cross-border malaria is a major challenge to elimination along the northern border of Brazil. Distinct demographic and socioeconomic profiles are unique to both border and transnational cases, which is critical to understand if malaria stratification measures are to be implemented effectively. Brazil’s goal of *Plasmodium falciparum* elimination by 2030 is intimately tied to the ability of the NMCP to stratify interventions based on risk. The third pillar of the WHO Global Technical Strategy for Malaria is to transform malaria surveillance into a core intervention as a means to achieve elimination. The results of this study demonstrate unique risk profiles for cross-border cases from Venezuela and Guyana in a mobility hotspot in the Brazilian Amazon, and speak to the importance of surveillance systems that quickly capture importation and thus inform mitigation strategies.

## Data Availability

Data utilized in the manuscript are publicly available through the Ministry of Health.

## Abbreviation

SIVEP: Malaria Epidemiological Surveillance Information System

## References

[1] Wangdi K, Gatton ML, Kelly GC, Clements ACA (2015). Cross-border malaria. A major obstacle for malaria elimination. Adv Parasitol..

[2] Steketee RAF, Alzahrani MH, Castro MC, Siqueira AM, Katabarwa MN, Xu JW (2018). Evidence review group on border malaria.

[3] Lover AA, Harvard KE, Lindawson AE, Smith Gueye C, Shretta R, Gosling R (2017). Regional initiatives for malaria elimination. Building and maintaining partnerships. PLoS Med.

[4] Saldanha R, Mosnier E, Barcellos C, Carbunar A, Charron C, Desconnets J-C (2020). Contributing to elimination of cross-border malaria through a standardized solution for case surveillance, data sharing, and data interpretation. Development of a cross-border monitoring system. JMIR Public Health Surveill..

[5] Douine M, Sanna A, Hiwat H, Briolant S, Nacher M, Belleoud D (2019). Investigation of a possible malaria epidemic in an illegal gold mine in French Guiana. An original approach in the remote Amazonian forest. Malar J..

[6] Hiwat H, Martínez-López B, Cairo H, Hardjopawiro L, Boerleider A, Duarte EC (2018). Malaria epidemiology in Suriname from 2000 to 2016. Trends, opportunities and challenges for elimination. Malar J..

[7] Ferreira MU, Castro MC (2016). Challenges for malaria elimination in Brazil. Malar J.

[8] Linn L, Lindmeier C. WHO certifies Paraguay malaria-free. Geneva, World Health Organization, 2018. https://www.whoint/news-room/detail/11-06-2018-who-certifies-paraguay-malaria-free.

[9] Burton RA, Chévez JER, Sauerbrey M, Guinovart C, Hartley A, Kirkwood G (2018). Factors associated with the rapid and durable decline in malaria incidence in El Salvador, 1980–2017. Am J Trop Med Hyg.

[10] WHO. World malaria report (2020). 20 years of global progress and challenges.

[11] Conn JE, Grillet ME, Correa M, Sallum MAM. Malaria Transmission in South America—Present Status and Prospects for Elimination. In: Manguin S, Dev V, Eds. Towards malaria elimination - a leap forward. InTech Open; 2018; pp. 281–313.

[12] Ferreira MU, Castro MC (2019). Malaria situation in Latin America and the Caribbean. Residual and resurgent transmission and challenges for control and elimination. Methods Mol Biol..

[13] Grillet ME, Hernández JV, Llewellyn MS, Paniz-Mondolfi A, Tami A, Vincenti-Gonzalez MF (2019). Venezuela’s humanitarian crisis, resurgence of vector-borne diseases and implications for spillover in the region. A call for action. Lancet Inf Dis..

[14] PAHO. Report on the situation of malaria in the Americas, 2017. Washington, Pan American Health Organization, 2017.

[15] United Nations Office for the Coordination of Humanitarian Affairs (OCHA). Humanitarian response plan with humanitarian needs overview Venezuela. 2020.

[16] Vreden SGS, Jitan JK, Bansie RD, Adhin MR (2013). Evidence of an increased incidence of day 3 parasitaemia in Suriname. An indicator of the emerging resistance of Plasmodium falciparum to artemether. Mem Inst Oswaldo Cruz..

[17] Vosti SA (1990). Malaria among gold miners in southern Para, Brazil. Estimates of determinants and individual costs. Soc Sci Med..

[18] Castellanos A, Chaparro-Narváez P, Morales-Plaza CD, Alzate A, Padilla J, Arévalo M (2016). Malaria in gold-mining areas in Colombia. Mem Inst Oswaldo Cruz.

[19] Moreno JE, Rubio-Palis Y, Paez E, Perez E, Sanchez V (2007). Abundance, biting behaviour and parous rate of anopheline mosquito species in relation to malaria incidence in gold-mining areas of southern Venezuela. Med Vet Entomol.

[20] da Franco CV, Peiter PC, Carvajal-Cortés JJ, Pereira DSR, Gomes MMDS, Suárez-Mutis MC (2019). Complex malaria epidemiology in an international border area between Brazil and French Guiana. Challenges for elimination. Trop Med Health..

[21] Musset L, Pelleau S, Girod R, Ardillon V, Carvalho L, Dusfour I, Gomes MS, Djossou F, Legrand E (2014). Malaria on the Guiana Shield. A review of the situation in French Guiana. Mem Inst Oswaldo Cruz..

[22] Doocy S, Page KR, de la Hoz F, Spiegel P, Beyrer C (2019). Venezuelan migration and the border health crisis in Colombia and Brazil. J Migration Hum Security.

[23] Grillet ME, Villegas L, Oletta JF, Tami A, Conn JE (2018). Malaria in Venezuela requires response. Science.

[24] Recht J, Siqueira AM, Monteiro WM, Herrera SM, Herrera S, Lacerda MVG (2017). Malaria in Brazil, Colombia, Peru and Venezuela current. Challenges in malaria control and elimination. Malar J..

[25] Sturrock HJW, Roberts KW, Wegbreit J, Ohrt C, Gosling RD (2015). Tackling imported malaria. An elimination endgame. Am J Trop Med Hyg..

[26] WHO Global Malaria Programme (2018). Malaria terminology.

[27] Brasil, Ministério da Saúde. Guia de Vigilância em Saúde. pp. 773. Brasília. Ministério da Saúde, Secretaria de Vigilância em Saúde, Coordenação-Geral de Desenvolvimento da Epidemiologia em Serviços. http://bvsms.saude.gov.br/bvs/publicacoes/guia_vigilancia_saude_1ed_atual.pdf; 2016.

[28] Ripley BD (2001). The R project in statistical computing. MSOR Connections The newsletter of the LTSN Maths, Stats & OR Network.

[29] Grillet ME, Moreno JE, Hernandez JV, Vincenti-Gonzalez MF, Noya O, Tami A, et al. Malaria in Southern Venezuela. The Hottest Hotspot in Latin America. bioRxiv 2020. 10.1371/journal.pntd.0008211 PMC7861532 33493212

[30] Goldani LZ (2018). Measles outbreak in Brazil, 2018. Braz J Inf Dis.

[31] Paniz-Mondolfi AE, Tami A, Grillet ME, Márquez M, Hernández-Villena J, Escalona-Rodríguez MA (2019). Resurgence of vaccine-preventable diseases in Venezuela as a regional public health threat in the Americas. Emerg Infect Dis.

[32] WHO (2015). Global technical strategy for malaria 2016–2030.

[33] Jianwei X, Hui L (1997). Border malaria in Yunnan, China. SE Asia J Trop Med Public Health.

[34] Khosa E, Kuonza LR, Kruger P, Maimela E (2013). Towards the elimination of malaria in South Africa. A review of surveillance data in Mutale Municipality, Limpopo Province, 2005 to 2010. Malar J..

[35] Ohrt C, Roberts KW, Sturrock HJ, Wegbreit J, Lee BY, Gosling RD (2015). Information systems to support surveillance for malaria elimination. Am J Trop Med Hyg.

[36] Nsanzabana C (2019). Strengthening surveillance systems for malaria elimination by integrating molecular and genomic data. Trop Med Infect Dis.

[37] Chenet SM, Akinyi Okoth S, Huber CS, Chandrabose J, Lucchi NW, Talundzic E (2016). Independent emergence of the *Plasmodium falciparum* kelch propeller domain mutant allele C580Y in Guyana. J Infect Dis.

[38] Mathieu LC, Cox H, Early AM, Mok S, Lazrek Y, Paquet J-C (2020). Local emergence in Amazonia of *Plasmodium falciparum* k13 C580Y mutants associated with in vitro artemisinin resistance. Elife.

[39] Brashear AM, Fan Q, Hu Y, Li Y, Zhao Y, Wang Z (2020). Population genomics identifies a distinct *Plasmodium vivax* population on the China-Myanmar border of Southeast Asia. PLoS Negl Trop Dis.

[40] Tessema SK, Raman J, Duffy CW, Ishengoma DS, Amambua-Ngwa A, Greenhouse B (2019). Applying next-generation sequencing to track falciparum malaria in sub-Saharan Africa. Malar J.

[41] Tessema S, Wesolowski A, Chen A, Murphy M, Wilheim J, Mupiri A-R (2019). Using parasite genetic and human mobility data to infer local and cross-border malaria connectivity in Southern Africa. Elife.

[42] Louzada J, de Almeida NCV, de Araujo JLP, Silva J, Carvalho TM, Escalante AA (2020). The impact of imported malaria by gold miners in Roraima. Characterizing the spatial dynamics of autochthonous and imported malaria in an urban region of Boa Vista. Mem Inst Oswaldo Cruz..

[43] Moreira JB, Baeninger R (2010). Local integration of refugees in Brazil. FMR..

[44] Douine M, Lambert Y, Musset L, Hiwat H, Blume LR, Marchesini P (2020). Malaria in Gold Miners in the Guianas and the Amazon. Current Knowledge and Challenges. Curr Trop Med Rep..

[45] Douine M, Sanna A, Galindo M, Musset L, de Santi VP, Marchesini P (2018). Malakit. An innovative pilot project to self-diagnose and self-treat malaria among illegal gold miners in the Guiana Shield. Malar J..

